# HIV-1 Induces DCIR Expression in CD4^+^ T Cells

**DOI:** 10.1371/journal.ppat.1001188

**Published:** 2010-11-11

**Authors:** Alexandra A. Lambert, Michaël Imbeault, Caroline Gilbert, Michel J. Tremblay

**Affiliations:** 1 Centre Hospitalier Universitaire de Québec-CHUL, Québec, Canada; 2 Département de Microbiologie-Infectiologie et Immunologie, Université Laval, Québec, Canada; NIH/NIAID, United States of America

## Abstract

The C-type lectin receptor DCIR, which has been shown very recently to act as an attachment factor for HIV-1 in dendritic cells, is expressed predominantly on antigen-presenting cells. However, this concept was recently challenged by the discovery that DCIR can also be detected in CD4^+^ T cells found in the synovial tissue from rheumatoid arthritis (RA) patients. Given that RA and HIV-1 infections share common features such as a chronic inflammatory condition and polyclonal immune hyperactivation status, we hypothesized that HIV-1 could promote DCIR expression in CD4^+^ T cells. We report here that HIV-1 drives DCIR expression in human primary CD4^+^ T cells isolated from patients (from both aviremic/treated and viremic/treatment naive persons) and cells acutely infected *in vitro* (seen in both virus-infected and uninfected cells). Soluble factors produced by virus-infected cells are responsible for the noticed DCIR up-regulation on uninfected cells. Infection studies with Vpr- or Nef-deleted viruses revealed that these two viral genes are not contributing to the mechanism of DCIR induction that is seen following acute infection of CD4^+^ T cells with HIV-1. Moreover, we report that DCIR is linked to caspase-dependent (induced by a mitochondria-mediated generation of free radicals) and -independent intrinsic apoptotic pathways (involving the death effector AIF). Finally, we demonstrate that the higher surface expression of DCIR in CD4^+^ T cells is accompanied by an enhancement of virus attachment/entry, replication and transfer. This study shows for the first time that HIV-1 induces DCIR membrane expression in CD4^+^ T cells, a process that might promote virus dissemination throughout the infected organism.

## Author Summary

The type II transmembrane protein DCIR belongs to the C-type lectin domain family receptor and is predominantly expressed in cells of the myeloid lineage. However recent evidence suggests that it can also be induced in CD4^+^ T cells placed under an inflammatory condition. We assessed the capacity of HIV-1 to promote DCIR expression in CD4^+^ T cells because the establishment of an inflammatory state is a hallmark of this retroviral infection in humans. We report here that a higher DCIR expression is detected not only in CD4^+^ T cells acutely infected with HIV-1 *in vitro* but also in clinical cell samples. Additional studies suggest a possible link between DCIR induction and apoptosis through both caspase-dependent and -independent intrinsic pathways. The greater expression of DCIR on the surface of CD4^+^ T cells results in more efficient virus attachment/entry, replication and transfer processes.

## Introduction

The Dendritic Cell ImmunoReceptor (DCIR) is a recently described member of the C-type lectin family. It is mainly expressed in cells of the myeloid lineage (i.e. neutrophils, dendritic cells, monocytes and macrophages) and also in B cells [1]. Its precise role and function are not completely understood but a recent work has suggested that DCIR might regulate expansion of dendritic cells (DCs) [2]. Moreover, it was previously established that DCIR can behave as an attachment factor for human immunodeficiency virus type-1 (HIV-1) on DCs and contribute possibly to virus dissemination by promoting both *cis*- and *trans*-infection processes [3]. Interestingly, DCIR is expressed on the surface of CD4^+^ T cells in rheumatoid arthritis (RA) patients before glucocorticoid treatment and a decrease of DCIR expression was seen with disease improvement [4]. This study provides the first indication that DCIR expression in CD4^+^ T cells can be promoted by inflammatory and immune hyperactivated conditions since RA is considered as a chronic, systemic inflammatory disorder characterized by a chronic T-cell response that has escaped normal control mechanisms [4], [5]. In addition, an increased surface expression of DCIR has been detected in patients suffering from a myocardial infarction [4], which corroborates that this molecule is induced by an inflammatory environment.

It is now well established that HIV-1 infection causes a slow but progressive impairment of the immune system, which is accompanied by a chronic hyperactivation of CD4^+^ and CD8^+^ T cells [6], [7], [8]. Consequently, infected patients display a heightened expression of various activation markers such as HLA-DR and CD38 in both CD4- and CD8-expressing T cells [9]. A relentless destruction of CD4^+^ T cells represents another hallmark of HIV-1 infection. The progressive loss of CD4^+^ T lymphocytes, either infected or uninfected (also called bystander), occurs through several distinct mechanisms. For example, it has been proposed that cell death is resulting from direct killing of virus-infected cells [10], elimination of HIV-1-infected CD4^+^ T cells by cytotoxic T lymphocytes (CTL) [11], syncytia formation through a gp120-mediated cell-to-cell fusion process [10], cytotoxic effects caused by some soluble viral proteins (e.g. Tat and Vpr) [10] and, finally, increased susceptibility to apoptosis in both infected and bystander cells that can be due, for example, to an interaction between the external envelope protein of HIV-1 (i.e. gp120) and primary cellular receptor/coreceptor (i.e. CD4 and CXCR4 or CCR5) [10], [12]. Importantly, previous studies suggest a direct correlation between the magnitude of apoptosis in circulating CD4^+^ T cells and disease pathogenesis [13], [14].

During evolution, the immune system has developed a number of strategies to fight viral infections, such as necrosis, autophagy and apoptosis. The last physiological mechanism is used by the body to eliminate overabundant cell populations and defective cells, and this form of cell death displays a propensity to be amplified and/or deregulated in various pathological processes [15]. Two major signalling pathways have been described to be involved in apoptosis induction, i.e. the intrinsic and extrinsic pathways. The first intracellular program is initiated by the disruption of the mitochondrial membrane and the release of mitochondrial proteins, such as cytochrome c, into the cytoplasm after developmental cues or severe cell stresses, such as DNA damage [16]. The extrinsic pathway is activated by the binding of ligands such as Fas ligand (FasL) (also termed CD95L), tumor necrosis factor (TNF), and TRAIL/Apo-2 ligand to their death receptors Fas/CD95/Apo-1, TNFR1 and DR4/DR5, respectively. These two pathways converge via activation of intracellular caspase-3 and -7. The caspase biochemical cascade ultimately triggers cell death through the destruction of cellular proteins and induction of DNA fragmentation [16]. It is known that apoptosis can also result from a caspase-independent process, which relies on the apoptosis-inducing factor (AIF). AIF represents the first mitochondrial protein shown to mediate cell death without the requirement for caspases. This protein is released from mitochondria and translocates to the nucleus, where it mediates nuclear features of apoptosis such as chromatin condensation and DNA degradation [17].

It has been shown that HIV-1 induces apoptosis in both infected and bystander immune effector cells [18], [19]. It has been established that at least five different virus-encoded proteins can induce apoptosis (i.e. Env, Tat, Nef, protease and Vpr) and three of them share a capacity to induce cell surface expression of death ligands and receptors of the TNF family (i.e. Env, Tat and Nef). Previous work indicates that HIV-1 induces a mitochondrial membrane permeabilization and release of AIF [20]. The regulatory protein Nef can confer protection against apoptosis but display also a converse capacity to induce apoptosis in neighbouring immune effector cells [21]. Cross-linking of CD4 molecules is the probable mechanism by which the virus-encoded gp120 can cause apoptosis in bystander CD4^+^ T cells [12], [22]. Moreover, the viral protease, which inactivates anti-apoptotic *Bcl-2* with a concomitant induction of the pro-apoptotic procaspase-8, renders the cell more prone to mitochondrial dysfunctions in response to internal or external death signals [23]. It has been proposed that apoptosis of bystander cells in the context of HIV-1 infection is likely to be multifactorial. Possible mechanisms include soluble factors secreted by HIV-1-infected cells as well as virus-encoded proteins (e.g. Env, Nef, TAT and Vpr) [24], [25]. For example, supernatants from HIV-1-infected DCs contain several heat labile soluble factors that mediate the killing of bystander thymocytes [26] and soluble factors were found to induce apoptosis in bystander cells [27], [28]. In addition, the viral accessory protein Vpr mediates apoptosis of bystander cells by causing the release of AIF [24].

Therefore given that RA and HIV-1 infection are both characterized by inflammatory and immune hyperactivation conditions and considering the recently described link between RA and DCIR expression in CD4+ T cells, we hypothesized that HIV-1 can trigger DCIR expression in CD4+ T cells.

## Results

### DCIR is up-regulated in CD4^+^ T cells from HIV-1-infected persons and following acute infection *in vitro*

**Figure 1:**
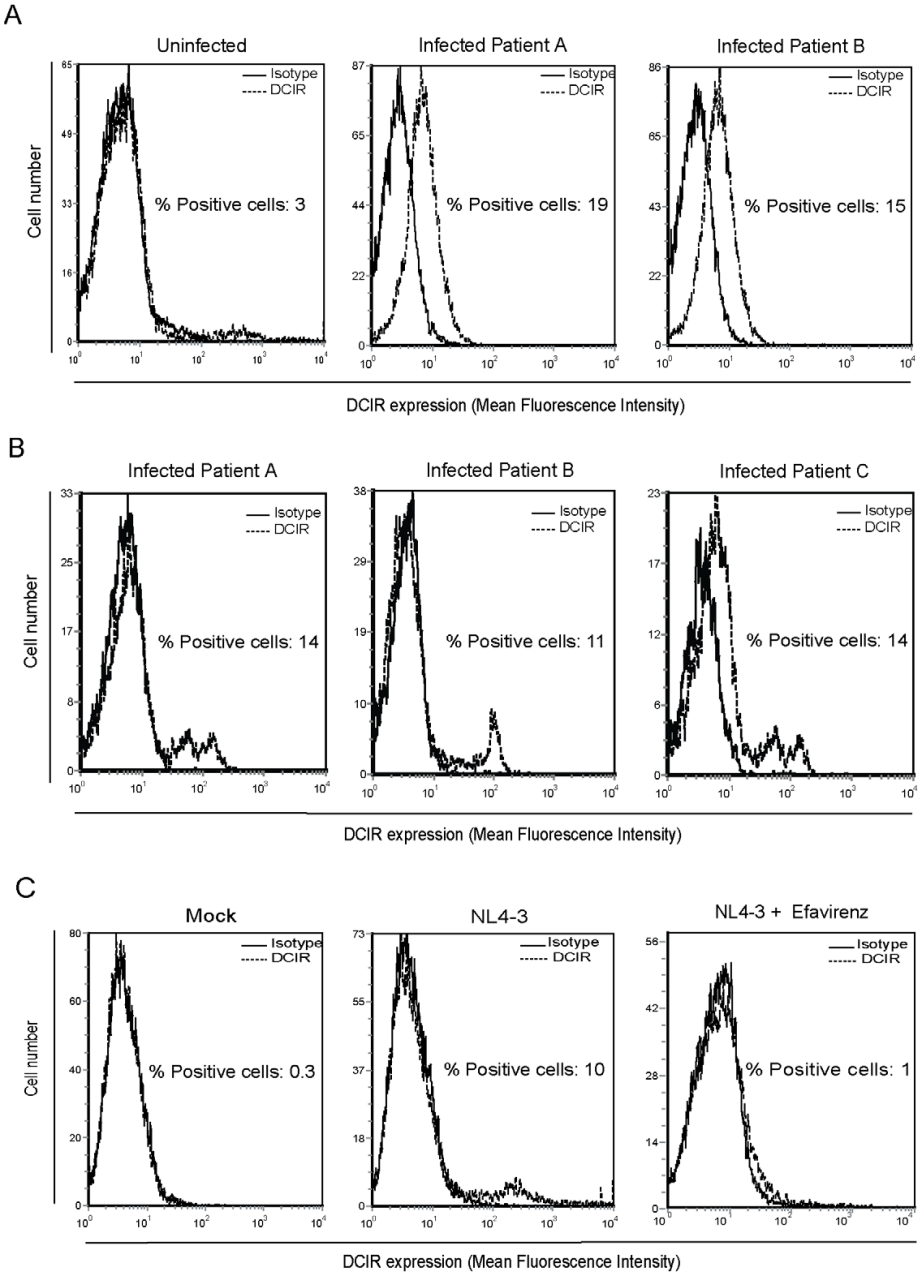
HIV-1 induces DCIR expression in CD4+ T cells under both *in vivo* and *in vitro* conditions. Purified CD4^+^ T cells were isolated from uninfected healthy donors and two HIV-1-infected aviremic/treated persons (P<0.05) (A) or three viremic/treatment-naive patients (P<0.001) (B). Next, cells (1×10^6^) were stained with the R-PE-labeled anti-DCIR monoclonal Ab. Expression of DCIR is shown as a dotted line, whereas the continuous line represents staining obtained with an isotype-matched irrelevant control Ab. For uninfected healthy donors, data shown correspond to a single experiment representative of 5 distinct donors. (C) Purified human primary CD4^+^ T cells (1×10^6^) were pulsed or not with NL4-3 (100 ng of p24). Three days later, DCIR expression was evaluated by flow cytometric analysis through the use of a R-PE-labeled anti-DCIR monoclonal Ab. Expression of DCIR is shown as a dotted line, whereas the continuous line represents results obtained with an isotype-matched irrelevant control Ab. Data shown in panel C correspond to a single experiment representative of 3 independent experiments (P<0.01). Statistical analyses were made by comparing fluorescence intensities in samples from HIV-1-infected patients or NL4-3-infected cells and the isotype-matched irrelevant control Ab.

DCIR has been detected in CD4^+^ T cells originating from patients with active RA, a chronic disease characterized by a state of persistent inflammation and immune activation. Because a systemic inflammatory disorder and immune hyperactivation represent also key features of the HIV-1 infection, we first assessed DCIR expression in CD4^+^ T cells isolated from infected individuals. To this end, the level of *ex vivo* DCIR expression was evaluated by flow cytometry in peripheral blood CD4^+^ T cells from two HIV-1-infected aviremic/treated patients. Results depicted in Figure 1A clearly indicate that DCIR is expressed in this cell subset in the context of a natural infection as opposed to what is seen in cells from uninfected healthy donors. Flow cytometry analyses were also performed on circulating CD4^+^ T cells from additional seropositive individuals but who were this time viremic and treatment-naive. Again an up-regulation of DCIR expression was detected in such samples (Figure 1B), which supports the concept that HIV-1 infection promotes expression of this C-type lectin receptor on the surface of circulating CD4^+^ T cells. A cell activation marker was also monitored as well (i.e. HLA-DR) and a positive correlation was found between DCIR and HLA-DR since both cell surface constituents were found to be increased in CD4^+^ T cells from viremic/treatment-naive persons compared to uninfected control samples (data not shown).

In an attempt to investigate further the capacity of HIV-1 to promote DCIR expression, *in vitro* studies were performed using human primary CD4^+^ T cells acutely infected with X4- and R5-using virus isolates (i.e. NL4-3 and NL4-3/Bal*env*, respectively). Exposure of purified CD4^+^ T cells to NL4-3 for 3 days triggers DCIR expression on the cell surface (Figure 1C). Similar observations were made when infection was carried out in parallel with the two tested viral isolates. For example, DCIR was detected in 9.0±1.5% and 8.6±0.8% of CD4^+^ T cells inoculated with NL4-3 and NL4-3/Bal*env*, respectively (n = 3) (data not shown). In some experiments, cells were first pre-treated with the antiretroviral drug efavirenz (EFV) before virus infection. This experimental strategy was used to decipher if the virus-mediated induction of DCIR requires a complete replicative cycle (i.e. productive infection). Treatment of purified CD4^+^ T cells with EFV reduced significantly the percentage of DCIR-expressing cells, thus indicating that productive infection with HIV-1 is mandatory to lead to DCIR expression. Altogether these results suggest that HIV-1 drives DCIR expression *in vivo* and *in vitro* in CD4^+^ T cells, a cell population recognized as a major cellular reservoir for HIV-1.

Experiments were also performed with Vpr- or Nef-deleted mutant to define the possible contribution of each single gene in the virus-mediated induction of DCIR expression on the surface of CD4^+^ T cells. Induction of DCIR was similar when cells were acutely infected with wild-type and Vpr- or Nef-deleted mutant viruses (data not shown).

### HIV-1 induces DCIR expression in both infected and bystander CD4^+^ T cells

**Figure 2:**
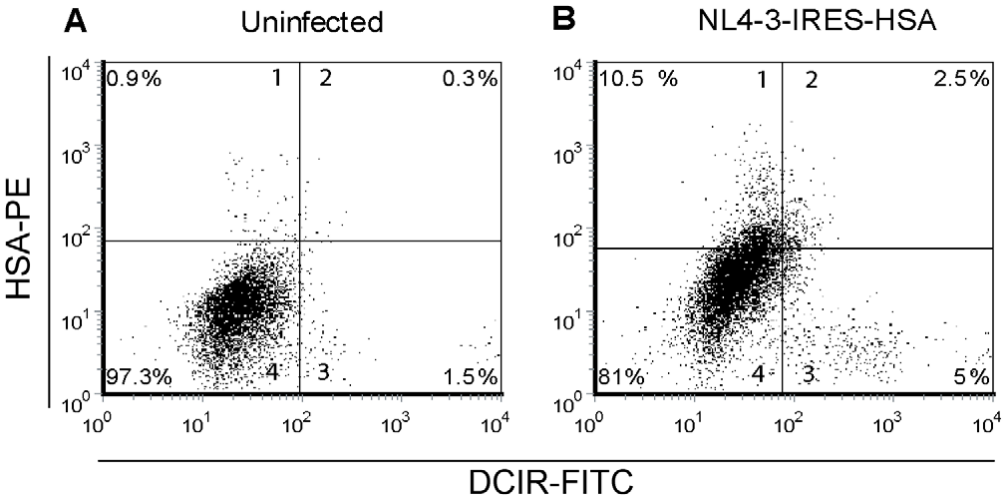
DCIR is expressed in both virus-infected and bystander CD4^+^ T cells. Cells (1×10^6^) were either left (A) uninfected or (B) infected with NL4-3-IRES-HSA reporter virus (100 ng of p24). Three days later, a double-stain flow cytometric method was performed to assess the percentages of DCIR-expressing and HSA-positive cells. Data shown correspond to a single experiment representative of 3 independent experiments.

We next set out to determine whether induction of DCIR occurs in virus-infected and/or bystander cells. This fundamental question was addressed through the use of a novel HIV-1 reporter construct, NL4-3-IRES-HSA, which, unlike most of the previous reporter constructs, will lead to the production of fully competent virions [29]. This X4-tropic infectious molecular clone of HIV-1 codes for all viral genes, with no deletions in *env*, *vpr*, or *nef*. It also expresses a cell surface reporter molecule, the murine heat-stable antigen (HSA), which permits the detection by flow cytometry of cells productively infected with HIV-1 through the surface expression of the HSA molecule. Briefly, human primary CD4^+^ T cells were exposed to NL4-3-IRES-HSA for 3 days and surface expression of HSA and DCIR was monitored by flow cytometry. Data shown in Table 1 demonstrate that 15.8±3.1% of cells are productively infected with HIV-1 (i.e. HSA-positive), whereas DCIR is expressed in 5.0±0.8% of cells and 2.3±0.2% of cells express both HSA and DCIR (n = 3) (a representative donor is depicted in Figure 2). Therefore, about 46% of DCIR-expressing cells are infected with HIV-1 and 56% of DCIR-positive cells are uninfected. It can be concluded that HIV-1 infection of CD4^+^ T cells promotes membrane expression of this C-type lectin surface receptor in both virus-infected and bystander cells.

**Table 1:** HIV-1 induces DCIR expression in CD4^+^ T cells^a^.

Cell surface marker	Mock	NL4-3-IRES-HSA
DCIR	1.0±0.2^b^	5.0±0.8
HSA	1.2±0.8	15.8±3.1
DCIR/HSA	0.6±0.5	2.3±0.2

a CD4+ T cells (1×10^6^) were either left uninfected (mock) or infected with NL4-3-IRES-HSA (100 ng of p24). Three days later, a double-staining method was used to estimate DCIR and HSA expression by flow cytometry.

b Data shown correspond to the means±SD of triplicate samples from 3 distinct donors.

### DCIR expression in bystander cells is due to soluble factors produced by CD4^+^ T cells productively infected with HIV-1

**Figure 3:**
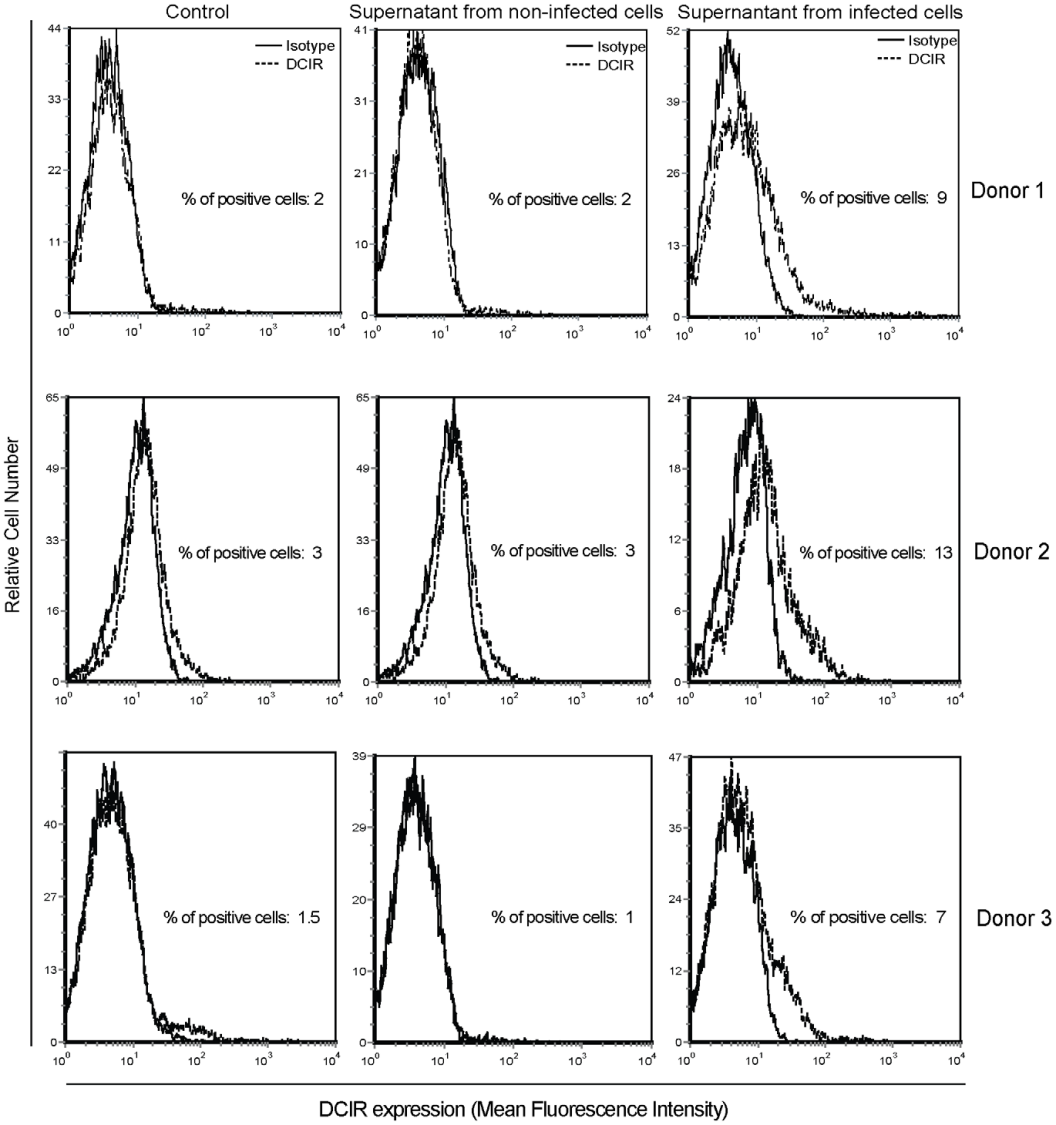
Soluble factors secreted by virus-infected cells promote DCIR expression. Cell-free supernatants from mock- and HIV-1-infected cells were used to treat purified CD4^+^ T cells. DCIR expression was monitored 3 days later by flow cytometry. Expression of DCIR is shown as a dotted line, whereas the continuous line represents staining obtained with an isotype-matched irrelevant control Ab. Data shown correspond to studies performed with three distinct donors.

Our previous findings indicate that HIV-1 induces DCIR expression not only in virus-infected but also in bystander cells as well. Our next set of experiments was aimed at defining the possible involvement of soluble factors produced by infected cells in the up-regulation of DCIR seen in bystander cells. To this end, human primary CD4^+^ T cells were cultured with cell-free culture supernatants from HIV-1-infected cells and DCIR expression was monitored by flow cytometry. As shown in Figure 3, exposure of CD4^+^ T cells to supernatants originating from cells acutely infected with HIV-1 is sufficient *per se* to drive DCIR expression in the three distinct donors studied.

### Correlation between HIV-1-mediated DCIR expression and apoptosis through both caspase-dependent and -independent intrinsic pathways

**Figure 4:**
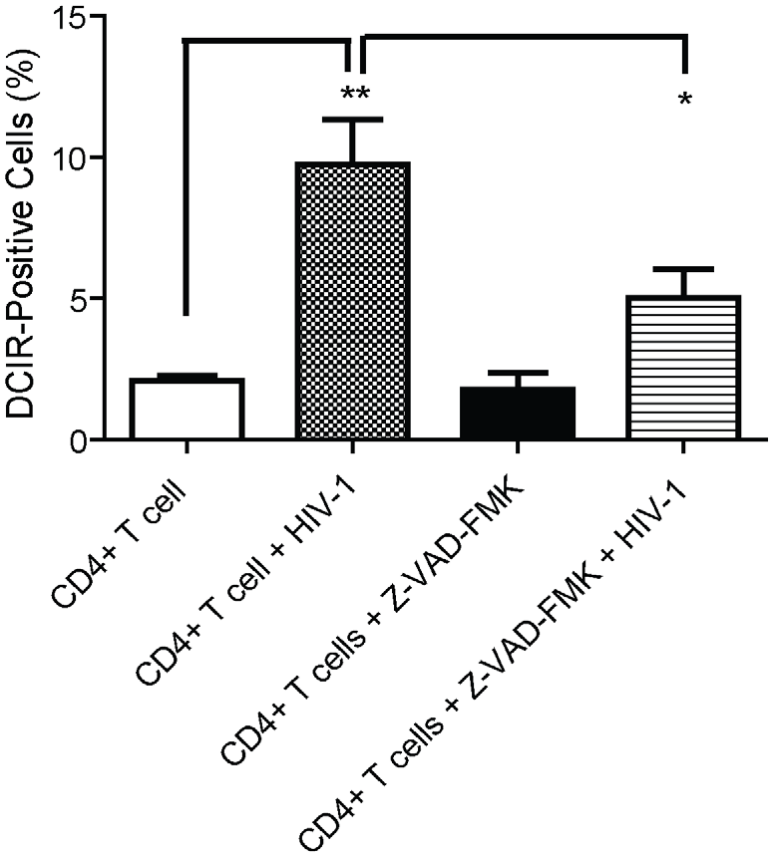
Virus-mediated induction of DCIR is partly prevented by a caspase inhibitor. Mitogen-activated CD4^+^ T cells (1×10^6^) were first either left untreated or treated for 1 h with the caspase inhibitor Z-VAD-FMK (50 nM), after which HIV-1 was added (100 ng of p24), where indicated. DCIR expression was monitored 3 days later by flow cytometry. Data shown represent the means ± SD of triplicate samples from three independent experiments. Asterisks denote statistically significant data (*, P<0.05; **, P<0.01).

The HIV-1-mediated induction of apoptosis in both infected and bystander CD4^+^ T cells is a well-described phenomenon [30], [31], [32]. The peak of apoptosis is observed usually after 2 to 3 days [20], the same time frame in which we detected the HIV-1-dependent induction of DCIR. Therefore, we next assessed whether there might be a connection between the virus-induced DCIR expression and apoptosis. We initially assessed the ability of NL4-3-IRES-HSA reporter virus to drive apoptosis in CD4^+^ T cells using FITC-VAD-FMK staining. This fluorochrome-labeled pan-caspase inhibitor is a specific and convenient-to-use marker of apoptotic cells, which can identify very early events of apoptosis associated with caspase activation (i.e. pre-apoptotic cells) [33]. Our studies indicate that NL4-3-IRES-HSA virions can potently mediate apoptosis in human primary CD4^+^ T cells (data not shown). As expected, the percentages of apoptotic cells in both virus-infected (i.e. HSA-positive) and bystander cells (HSA-negative) were significantly reduced upon EFV treatment (data not shown). To establish a link between DCIR expression and apoptosis following HIV-1 infection, we carried out a series of investigations with the broad-spectrum caspase inhibitor Z-VAD-FMK [34]. As illustrated in Figure 4, the HIV-1-mediated DCIR up-regulation was partially reduced in presence of Z-VAD-FMK, thus suggesting that the virus-directed increased expression of DCIR is associated with both caspase-dependent and -independent apoptotic pathways.

**Figure 5:**
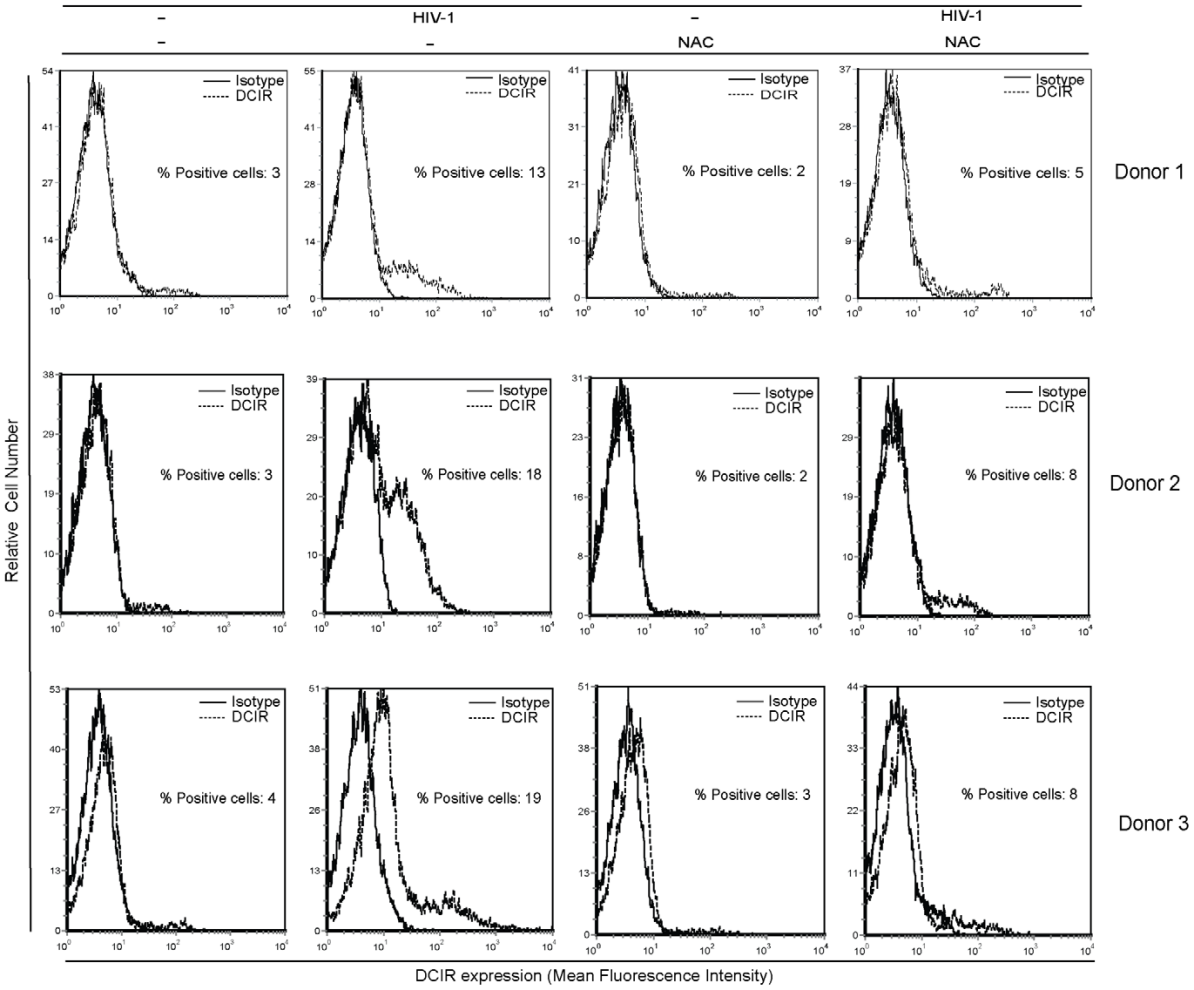
HIV-1-dependent DCIR induction is due also to a caspase-independent process involving AIF. Cells (1×10^6^) were first either left untreated or treated for 1 h with NAC, after which HIV-1 (100 ng of p24) was added. DCIR expression was monitored 3 days later by flow cytometry. Expression of DCIR is shown as a dotted line, whereas the continuous line represents staining obtained with an isotype-matched irrelevant control Ab. Data shown correspond to 3 independent experiments performed with distinct healthy donors.

To shed light on the nature of the caspase-independent death mechanism, we studied the involvement of the apoptotic effector protein AIF based on the previous report showing that HIV*-*1 induces a mitochondrial-mediated but caspase-independent apoptosis controlled by AIF [35]. The possible contribution of AIF was investigated through the use of the inhibitor of apoptosis N-acetyl-L-cystein (NAC), which blocks nuclear translocation of AIF. Our results demonstrate that the HIV-1-induced expression of DCIR on the surface of human primary CD4^+^ T cells is inhibited but not completely by a NAC treatment (i.e. 16.2±3.4% in HIV-1-infected cells compared to 6.7±1.7% in virus-infected cells also treated with NAC) (n = 3) (these three donors are depicted in Figure 5). Experiments were performed also with both Z-VAD-FMK and NAC to see if this double treatment can totally inhibit the virus-mediated induction of DCIR expression. Unfortunately the concomitant use of the two compounds is leading to cell toxicity (data not shown). It should be noted that no toxicity is seen when each compound are tested individually (data not shown). Nevertheless, we provide evidence that there is a close connection between DCIR expression and apoptosis (through caspase-dependent and -independent pathways) after acute infection of CD4^+^ T cells with HIV-1.

### HIV-1 infection of CD4^+^ T cells results in DCIR expression partly due to a free radical, caspase-dependent apoptosis pathway

**Figure 6:**
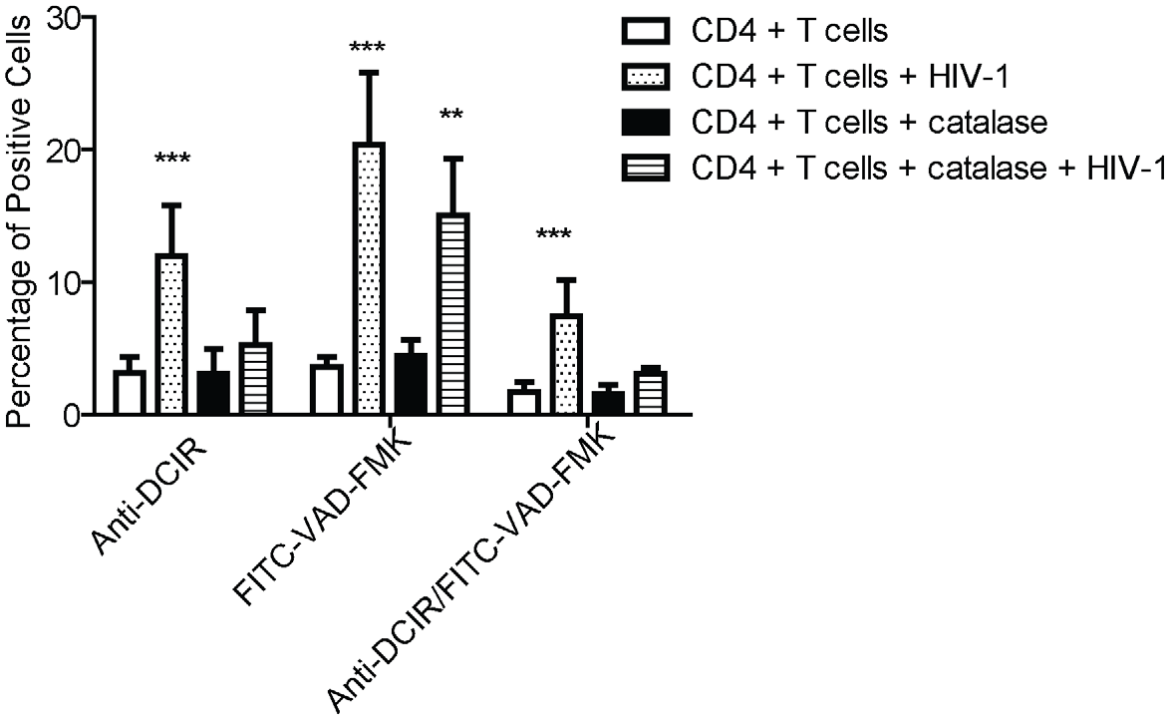
H_2_O_2_ produced by HIV-1-infected cells promotes DCIR expression. Mitogen-stimulated CD4^+^ T cells (1×10^6^) were either left untreated or treated with catalase before HIV-1 infection. Three days after virus infection, DCIR expression was measured by flow cytometry. Data shown represent the means ± SD of triplicate samples from three independent experiments. Asterisks denote statistically significant data (**, P<0.01; ***, P<0.001).

In HIV-1-infected patients, the hyperactivation status is accompanied by an increased production of free radicals (e.g. superoxide anion, hydroxyl radical and hydrogen peroxide). This excess of reactive oxygen species (ROS) damages cell membranes and generates apoptosis [36]. To establish a putative relationship between DCIR expression and apoptosis induced by free radicals after HIV-1 infection, we performed a double staining with anti-DCIR and FITC-VAD-FMK in virus-infected CD4^+^ T cells treated with catalase because this enzyme is a known scavenger of ROS (including hydrogen peroxide). Results depicted in Figure 6 suggest that free radicals are indeed playing a functional role in the HIV-1-mediated induction of DCIR seen in apoptotic cells (i.e. positive for both DCIR and FITC-VAD-FMK).

**Figure 7:**
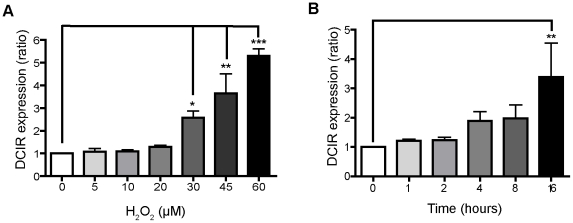
H_2_O_2_ mediates both apoptosis and DCIR expression. Mitogen-stimulated CD4^+^ T cells (1×10^6^) were exposed to increasing concentrations of H_2_O_2_ for 16 h (A) or treated with a constant dose of H_2_O_2_ (i.e. 30 µM) for the indicated time lengths (B). Next, DCIR expression was assessed by flow cytometry. Data shown represent the ratio of DCIR expression over basal expression. The ratio is calculated from the means ± SD of triplicate samples from three independent experiments. Asterisks denote statistically significant data (*, P<0.05; **, P<0.01; ***, P<0.001).

Hydrogen peroxide (H_2_O_2_), a representative ROS, has been extensively used to study apoptosis following an oxidative stress [37]. Thus, additional experiments were performed in human primary CD4^+^ T cells using H_2_O_2_ as an inducer of an apoptotic-like cell death. Exposure of mitogen-stimulated CD4^+^ T cells to concentrations of H_2_O_2_ ranging from 20 to 60 µM led to a dose-dependent increased in DCIR expression (Figure 7A). Cell viability was reduced when using doses of H_2_O_2_ ≥45 µM (data not shown). Consequently, the subsequent experiments were performed using H_2_O_2_ at a final concentration of 30 µM. A time-course analysis indicated that the H_2_O_2_-mediated expression of DCIR is maximal at 16 h post-treatment and reached a plateau at a longer time period (i.e. 32 h) (Figure 7B and data not shown). The specificity of the relation between DCIR expression and apoptosis was addressed by estimating surface expression of two other HIV-1 receptors, namely DC-SIGN (used as a negative control) and CD4. Our data demonstrate that both cell surface molecules are not modulated upon induction of apoptosis by H_2_O_2_ (data not shown). Importantly, DCIR was promoted as well by staurosporine (data not shown), a well-known inducer of apoptosis in a wide range of cell lines [38], which further confirms the connection between DCIR and apoptosis.

**Figure 8:**
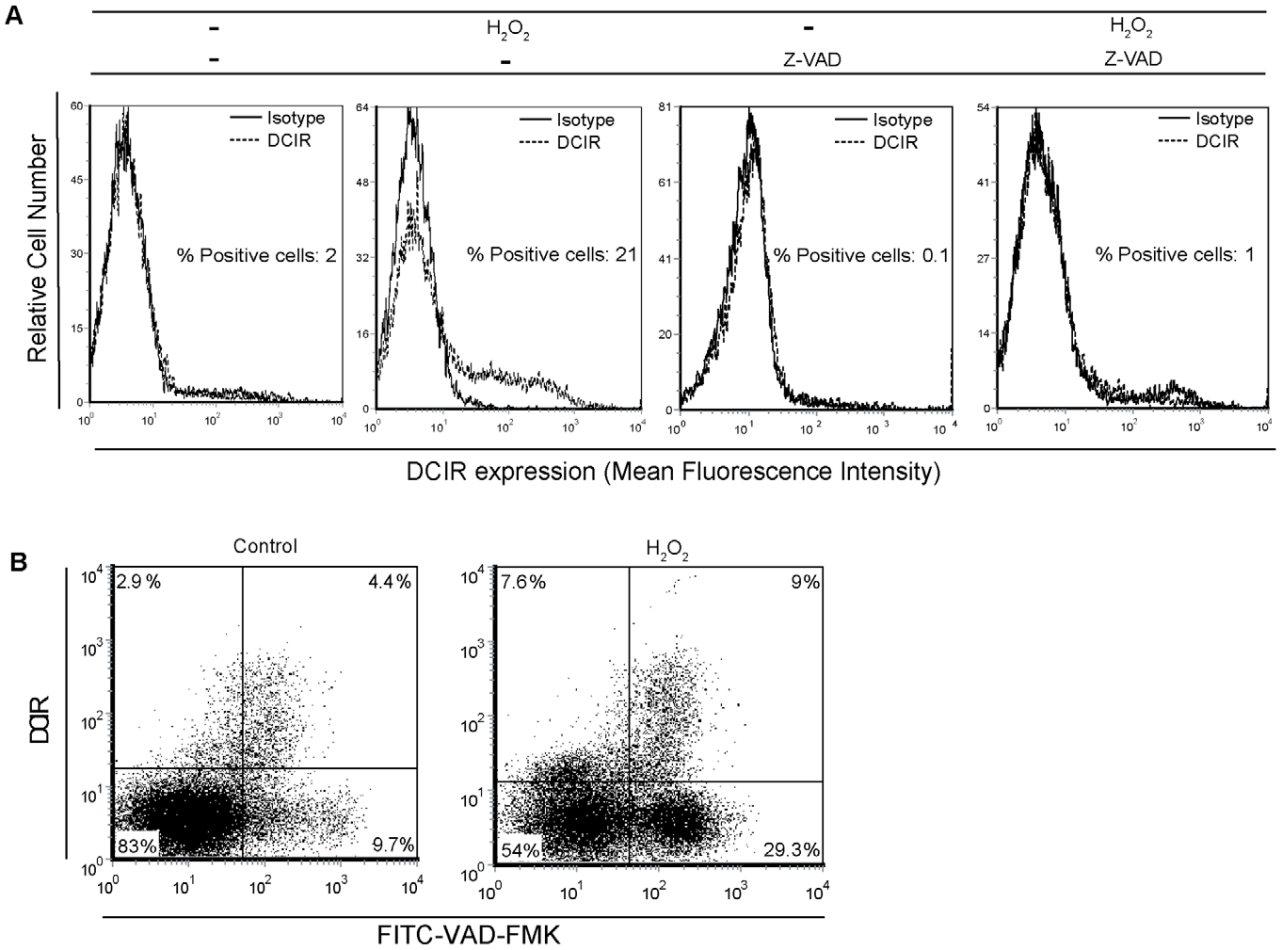
H_2_O_2_ treatment drives DCIR expression in both nonapoptotic and apoptotic cells. (A) Mitogen-activated CD4^+^ T cells (1×10^6^) were first either left untreated or treated for 1 h with the caspase inhibitor Z-VAD-FMK (50 nM), after which H_2_O_2_ (30 µM) was added, where indicated. DCIR expression was monitored 16 h later by flow cytometry. Expression of DCIR is shown as a dotted line, whereas the continuous line represents staining obtained with an isotype-matched irrelevant control Ab. Data shown correspond to a single experiment representative of 3 independent experiments. (B) Mitogen-stimulated CD4^+^ T cells (1×10^6^) were first treated for 16 h with H_2_O_2_ (i.e. 30 µM). Thereafter, DCIR surface expression and caspase activation were monitored by flow cytometric analysis using a double-staining method consisting of FITC-VAD-FMK followed by the R-PE-conjugated anti-DCIR. Data shown correspond to a single experiment representative of 4 independent experiments.

Given that H_2_O_2_ induces also necrosis and mediates apoptosis primarily via a caspase-dependent pathway [39], [40], we performed experiments with Z-VAD-FMK. A pre-treatment with Z-VAD-FMK prevented DCIR expression in activated CD4^+^ T cells after H_2_O_2_ stimulation (i.e. 21.4±3.4% in H_2_O_2_-treated cells compared to 1.0±0.2% in cells treated with both H_2_O_2_ and Z-VAD-FMK) (n = 3) (a representative donor is depicted in Figure 8A). Experiments were repeated in quiescent CD4^+^ T cells and we made similar observations (data not shown). Overall our results indicate that the H_2_O_2_-driven induction of DCIR is not due to necrosis and occurs through a caspase-mediated signal transduction pathway. Moreover, we estimated the percentages of apoptotic cells that express DCIR following H_2_O_2_ treatment. For this purpose, human primary CD4^+^ T cells were labelled with FITC-VAD-FMK and anti-DCIR. We found that 12.2±3.2% of apoptotic cells are also positive for DCIR (n = 3) (a representative donor is depicted in Figure 8B), which confirms the relationship between DCIR and apoptosis in CD4^+^ T cells.

### HIV-1 binding/entry, infection and transfer processes are all promoted by ROS-mediated induction of DCIR

**Figure 9:**
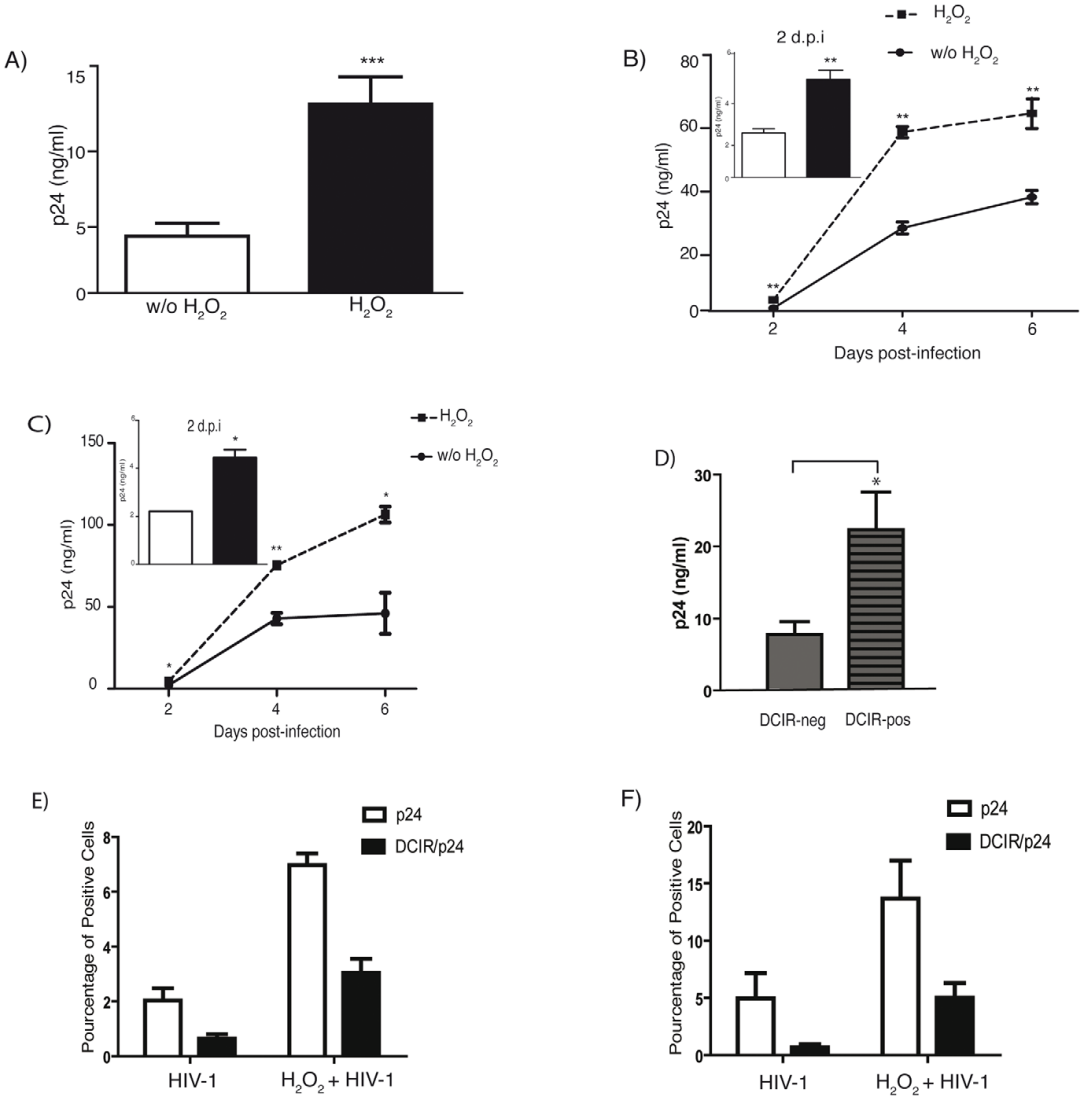
HIV-1 attachment/entry, replication and transfer processes are all promoted in H_2_O_2_-treated CD4^+^ T cells. Target CD4^+^ T cells (1×10^6^) were treated for 16 h with H_2_O_2_ (30 µM) to induce surface expression of DCIR. (A) Cells were next exposed to NL4-3 (100 ng of p24) for 1 h at 37°C, extensively washed to remove unabsorbed virons before assessing the p24 content. (B) Cells were first incubated with NL4-3 (100 ng of p24) for 2 h at 37°C, washed extensively to remove input virus and cultured in complete culture RPMI-1640 medium supplemented with rhIL-2 for the indicated number of days. Cell-free supernatants were collected and assayed for the p24 content. (C) Cells were exposed to NL4-3 (100 ng of p24) for 2 h at 37°C, next washed extensively to remove input virus, and finally co-cultured with autologous CD4^+^ T cells in complete culture RPMI-1640 medium supplemented with rhIL-2 for the indicated number of days. Cell-free supernatants were collected and assayed for the p24 content. Virus production at day 2 is depicted in the small inserts (panels B and C). (D) Cells were exposed to NL4-3 (100 ng of p24) for 2 h at 37°C, washed extensively to remove input virus, and maintained in complete culture medium supplemented with rhIL-2 for 3 days. Next, DCIR-negative and -positive cells (used as transmitter cells) were isolated with magnetic beads and co-cultured with uninfected CD4^+^ T cells (used as recipient cells). Cell-free supernatants were collected at 3 days following initiation of the co-culture and assayed for the p24 content. (E) Cells were first exposed to NL4-3 for 2 h at 37°C. Cells were extensively washed to remove unabsorbed virions and half of the cells were used to estimate the percentage of cells positive for surface DCIR and intracellular p24. (F) The other half was maintained for 3 days in culture before assessing both DCIR and p24. Data shown represent the means±SD of triplicate samples and correspond to a single experiment representative of three independent experiments. Asterisks denote statistically significant data (*, P<0.05; **, P<0.01; ***, P<0.001).

Taken together, our findings demonstrated that the HIV-1-mediated apoptosis promotes DCIR surface expression in CD4^+^ T cells. Previous results indicate that DCIR can capture HIV-1 on DCs, enhance *de novo* virus production by DCs (i.e. infection *in cis*), and increase DC-mediated virus transmission to CD4^+^ T cells (i.e. infection *in trans*) [3]. Experiments were thus carried out to define first whether HIV-1 attachment/entry in CD4^+^ T cells can be affected by the H_2_O_2_-dependent increase in DCIR expression. As illustrated in Figure 9A, the early steps in the virus life cycle (i.e. binding and entry) are enhanced in CD4^+^ T cells following exposure to H_2_O_2_ (i.e. 12.5±1.8 versus 3.8±0.8 ng/ml of p24). We next set out to determine whether acute HIV-1 infection was also affected under these conditions. A statistically significant increase in virus production was seen in cells treated with H_2_O_2_ (Figure 9B). Similarly, virus transfer was also enhanced when DCIR-expressing CD4^+^ T cells are used as transmitter cells (Figure 9C). To further strengthen the contribution of DCIR in the virus *trans*-infection pathway, CD4^+^ T cells were first exposed to H_2_O_2_ to induce DCIR expression. Thereafter, DCIR-negative and DCIR-positive cells were isolated and used separately in HIV-1 transfer experiments. Data shown in Figure 9D demonstrate that HIV-1 transmission toward uninfected CD4^+^ T cells (i.e. recipient cells) is augmented when using, as transmitter cells, DCIR-positive CD4^+^ T cells. Finally, to substantiate the participation of DCIR in HIV-1 replication, H_2_O_2_-treated/virus-infected CD4^+^ T cells were subjected to a dual staining immunofluorescence method to detect both intracellular HIV-1 p24 and surface DCIR. An increase in virus binding/entry was detected in H_2_O_2_-treated cells expressing DCIR (Figure 9E). A similar augmentation in cells expressing both DCIR and p24 was detected following acute virus infection (Figure 9F).

## Discussion

It has been already recognized that neutrophils, monocytes, DCs, macrophages and B cells constitutively express a high level of DCIR, as opposed to CD4^+^ T cells that are negative for DCIR [1]. However, data from a recent work suggest that this type II membrane glycoprotein is expressed on CD4^+^ T cells in RA patients and the level of DCIR surface expression is higher in the rheumatic joint compared to peripheral blood [4]. Since DCIR expression is reduced following a local corticosteroid treatment, it was proposed that there is a potential connection between an inflammatory state and DCIR expression [4]. We established previously that DCIR can serve as an attachment factor for HIV-1 [3], which is the causative agent of AIDS, another disease characterized by a chronic inflammatory state. Starting from these initial intriguing observations, we monitored DCIR expression on the surface of circulating CD4^+^ T cells isolated from HIV-1-carrying patients. We report here that DCIR is expressed on CD4^+^ T cells originating from aviremic/treated and viremic/untreated seropositive patients, whereas, no expression was detected in cells from healthy donors. These results suggest that DCIR expression on the surface of circulating CD4^+^ T cells seems to be a generalized phenomenon in the context of various inflammatory diseases.

To acquire additional information about the ability of HIV-1 to induce DCIR expression in a cell subpopulation that is infected under physiological conditions, we performed *in vitro* experiments where human primary CD4^+^ T cells were acutely infected with X4- and R5-tropic virions and monitored DCIR expression. We showed that HIV-1 drives DCIR expression in both infected and bystander cells. Moreover, we monitored DCIR levels in the CD4^+^ T cell subpopulation following acute HIV-1 infection of unseparated peripheral blood mononuclear cells. Unfortunately, no conclusive data could be obtained because we detected a high mortality rate probably due to the presence of CD8^+^ T cells. In HIV-1 infection, disease progression correlates with elevated levels of apoptosis [13]. Therefore, we defined whether expression of the immunoreceptor DCIR on the surface of CD4^+^ T cells in the context of HIV-1 infection could perhaps be considered as a possible marker of apoptosis for these cells. We performed experiments and discovered effectively that there is a certain correlation between HIV-1 infection, DCIR expression and induction of apoptosis. We provide evidence that there is a connection between HIV-1-mediated induction of DCIR expression and apoptosis, the latter being caused by a caspase-dependent pathway in response possibly to a mitochondrial H_2_O_2_ generation by virus-infected cells and a caspase-independent process involving AIF. Our data are in agreement with published reports since Vpr has been shown to induce cell death via the mitochondrial caspase-independent death effector AIF [35] and Vpr can also induce a decrease of mitochondrial membrane potential along with the release of cytochrome c [41].

HIV-1 infection induces prolonged immune system activation that may cause local or systemic oxidative stress and thus result in oxidative damage. Oxidative stress occurs when the balance of antioxidant protection and the production of free radicals, primarily reactive oxygen and nitrogen molecules are disturbed [42], [43]. Some viral proteins play a role in the intracellular increase of ROS (e.g. superoxide anion, hydroxyl radical and hydrogen peroxide) which in turn influence the increase in the apoptotic index causing a decrease of CD4^+^ T cells and more importantly increase in HIV-1 replication secondary to free radicals overproduction [42]. This excess of ROS damages cell membranes and generates apoptosis [36]. This may account for the loss of CD4^+^ T cells seen during progression of HIV-1 infection toward AIDS. Oxygen radicals produced under circumstances that occur during opportunistic infections mediate apoptosis and this effect is reversed by oxygen radical scavengers [44]. We corroborated that human primary CD4^+^ T cells are sensitive to apoptosis caused by H_2_O_2_, a representative ROS that has been extensively used to study apoptosis following an oxidative stress [37]. Based on this information and the previous demonstration that free radicals are actively produced by CD4^+^ T cells from HIV-1-carrying patients [36], we showed here that H_2_O_2_ induces also DCIR expression. The H_2_O_2_-mediated induction of apoptosis was not only detected in human primary CD4^+^ T cells but also in Raji and 293T cells (data not shown). No increase in DCIR expression was seen when using a previously reported anti-Fas monoclonal antibody [45] (data not shown), which is an effector of the extrinsic apoptosis pathway [46]. These results indicate that the DCIR induction in CD4^+^ T cells seen after HIV-1 infection is partly associated with a caspase-dependent intrinsic apoptotic process.

An increased expression of DCIR was also observed in a proportion of bystander cells undergoing apoptosis. Experiments carried out with cell-free supernatants from HIV-1-infected cells revealed that soluble factors are sufficient to drive not only apoptosis but also DCIR expression. The phenomenon of bystander cell apoptosis is well described in the literature. Indeed, numerous viral proteins have been described as responsible for causing apoptosis in bystander cells (e.g. Env, Nef, TAT and Vpr) [24]. Supernatants from HIV-1-infected DCs contain several heat labile soluble factors that cause cell death in bystander thymocytes [26] and soluble factors were shown to induce apoptosis in bystander cells [27], [28]. The transactivating protein Tat is released in the surrounding microenvironment and can be taken up by neighbouring bystander cells, which will ultimately undergo apoptosis [28], [47]. Vpr has been detected in sera and cerebrospinal fluid from HIV-1-infected patients [48], [49] and this protein of viral origin is recognized as a potent inducer of cell death via a caspase-independent mitochondrial pathway [24], [35]. Recently, Lenassi and co-workers established that Nef induces the release of exosomes from T cells, which transport extracellular Nef and cause apoptosis of bystander CD4^+^ T cells [50]. Surprisingly, studies with Nef- or Vpr-deleted mutants suggest that these two viral genes are not involved in the HIV-1-mediated induction of DCIR expression in CD4^+^ T cells. Therefore, it can be proposed that the virus-directed induction of DCIR and apoptosis is caused by a multifactorial phenomenon that needs to be identified.

More relevant to the pathogenesis of HIV-1 infection, we demonstrated that the H_2_O_2_-mediated induction of DCIR and apoptosis is coupled with an increased virus binding/entry and higher replication of HIV-1 in CD4^+^ T cells. Additionally, the noticed up-regulated DCIR expression is also leading to more efficient virus propagation. It can be proposed that DCIR, once expressed onto such CD4^+^ T cells, can participate actively to HIV-1 propagation. Although it might seem irrational that apoptotic cells would be more susceptible to productive HIV-1 infection, it should be stated that the fluorochrome-labeled pan-caspase inhibitor FITC-VAD-FMK, which was used to monitor the link between HIV-1-mediated DCIR expression and apoptosis, permits to identify the very early events of apoptosis (i.e. pre-apoptotic cells). Thus it is possible that virus binding/entry and replication processes can still occur during a certain time period in CD4^+^ T cells that are in a pre-apoptotic state. It is known that HIV-1 exploits different strategies to escape the immune response including a rapid/high mutation rate, down-regulation of major histocompatibility complex class-I molecules, broad coreceptor usage and destruction of both CD4- and CD8-expressing T cells [51]. We suggest that HIV-1 can utilize DCIR as another tactic for escaping the immune system and/or increasing its infectivity. Different hypotheses may be formulated with respect to the role(s) played by DCIR once expressed on the surface of CD4^+^ T cells.

**Figure 10:**
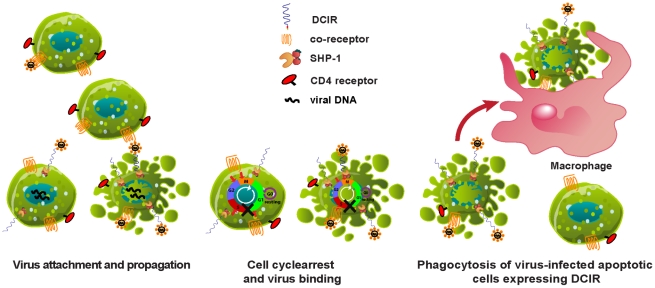
Proposed working models for DCIR involvement in HIV-1 infection. DCIR expression is promoted not only in cells productively infected with HIV-1 but also in bystander cells via both a mitochondrial (intrinsic) caspase-dependent apoptotic pathway and a caspase-independent apoptotic process relying on AIF. The resulting DCIR induction on the surface of CD4^+^ T cells can affect virus replication by various means. For example, virus binding can be increased through DCIR, a process leading to a more efficient HIV-1 propagation. Moreover, the cell cycle arrest seen in DCIR-expressing cells can also promote virus attachment and the ensuing HIV-1 transmission despite apoptosis induction because of the association between DCIR and SHP-1. It can also be postulated that DCIR expression on the surface of apoptotic CD4^+^ T cells also infected with HIV-1 might facilitate phagocytosis by macrophages and DCs, thereby favoring infection of such antigen-presenting cells and viral propagation.

It can be hypothesized that induction of apoptosis increases virus attachment/entry likely through DCIR expression on the surface of CD4^+^ T cells (Figure 10). This theory is supported by our results showing that the H_2_O_2_-mediated induction of apoptosis in CD4^+^ T cells and DCIR expression are not accompanied by a modulation of surface expression of two other attachment factors for HIV-1, i.e. DC-SIGN and CD4. DCIR carries an immunoreceptor tyrosine-based inhibitory motif (ITIM) in its cytoplasmic tail that is thought to be responsible for the immunoregulatory role played by this cell surface molecule. The intracellular ITIM motif of DCIR is involved in SHP-1 recruitment [52], a tyrosine phosphatase known for its important role in maintaining cellular homeostasis [53]. The protein tyrosine phosphatase SHP-1 has also been shown to regulate HIV-1 transcription [54] and inhibit antigen-receptor-induced apoptosis [55]. Interestingly, DCIR-expressing cells following acute HIV-1 infection display a cell cycle arrest (data not shown), which might permit virus attachment despite the appearance of a pre-apoptotic state. Studies are currently performed to address this possibility. Thus, the life cycle of HIV-1 can be affected in several ways by the newly expressed DCIR and recruited SHP-1 molecules.

It can also be postulated that DCIR expression may lead to phagocytosis by macrophages and DCs of apoptotic CD4^+^ T cells also infected with HIV-1, thereby promoting viral propagation and infection of such antigen-presenting cells. It is well established that macrophages play a central role in the pathogenesis of HIV-1 infection, functioning as stable viral reservoir due to their ability to resist HIV-1-mediated cytopathicity. Of importance to note is the previous report showing that phagocytosis of apoptotic cells induced an increase in HIV-1 replication in macrophages [56]. Similarly, we observed that HIV-1 replication in macrophages is enhanced when such cells are co-cultured with DCIR-positive apoptotic CD4^+^ T cells treated with H_2_O_2_ (data not shown).

Additionally, instead of inducing an inflammatory immune response, phagocytosis of DCIR-expressing apoptotic cells might promote the generation of suppressor macrophages as described previously for bacterial infections [57] and tumor cells [58]. This would allow microorganisms such as HIV-1 to escape the immune system. Alternatively, it is possible that DCIR is induced after HIV-1 infection because it acts as a death signal for the cell and/or as a sign to promote phagocytosis. It can also be proposed that DCIR facilitates HIV-1 attachment before cell death, a process leading to more extensive virus dissemination across the organism. Supplementary experiments are warranted to validate these non-mutually exclusive hypotheses.

Together, our work represents the first evidence that DCIR can serve as a marker for apoptosis in the context of an HIV-1 infection. Additional studies are needed to define more firmly whether there is a connection between the chronic inflammatory state seen in HIV-1-infected persons and DCIR expression in CD4^+^ T cells. Importantly, the exact contribution of the immunoreceptor DCIR to HIV-1 pathogenesis needs to be delineated because it might provide novel therapeutic avenues.

## Materials and Methods

### Reagents

Recombinant human interleukin-2 (rhIL-2) and the non-nucleoside reverse transcriptase inhibitor EFV were obtained from the AIDS Repository Reagent Program (Germantown, MD). The mitogenic agent phytohemagglutinin-L (PHA-L) was purchased from Sigma (St-Louis, MO). The culture medium for human primary CD4^+^ T cells consisted of RPMI-1640 supplemented with 10% foetal bovine serum (FBS), penicillin G (100 U/ml), streptomycin (100 U/ml), glutamine (2 mM), which were all purchased from Wisent (St-Bruno, QC), and primocine, obtained from Amaxa Biosystems (Gaithersburg, MD). The culture medium for 293T cells was made of Dulbecco's modified Eagle's medium (DMEM) supplemented with 10% FBS and penicillin G (100 U/ml), streptomycin (100 U/ml), and glutamine (2 mM) (Invitrogen, Burlington, Canada).

### Antibodies

R-Phycoerythrin (R-PE)-conjugated and fluorescein isothiocyanate (FITC)-labelled anti-DCIR monoclonal antibodies (clone 216110) and the corresponding isotype-matched irrelevant control antibody (Ab) were purchased from R&D Systems (Minneapolis, MN). The FITC-labelled anti-DC-SIGN monoclonal antibody (Ab) (clone eB-h209) and the appropriate control Ab were purchased from eBioscience (San Diego, CA). R-PE-labelled anti-HSA Ab (clone M1/69) was purchased from Invitrogen (Burlington, USA), whereas biotin-tagged anti-HSA (clone M1/69) was purchased from BD Biosciences (Mississauga, ON). PE-Cy5 anti-streptavidin Ab was obtained from eBioscience and the FITC-conjugated anti-p24 from Beckman Coulter (Mississauga, ON).

### Production of viral stocks

Virions were produced upon transient transfection of human embryonic kidney 293T cells as previously described [59]. The infectious molecular clones used in this study included pNL4-3/Bal*env* (R5-tropic), pNL4-3 (X4-tropic) and its derivative pNL4-3-IRES-HSA (X4-tropic). The pNL4-3-IRES-HSA molecular construct was obtained by replacing the enhanced green fluorescent protein (*eGFP*) gene in the NLENG1-IRES vector (kindly supplied by D.N. Levy, New York University College of Dentistry, New York, NY) [60] with the coding sequence for mouse heat stable antigen (HSA) [29]. Experiments were also carried out with NL4-3-based mutant deleted in Nef (kindly supplied by S. Venkatesan, National Institute of Allergy and Infectious Diseases, Bethesda, MD) or Vpr (kindly provided by E.A. Cohen, Institut de Recherches Cliniques de Montréal, Montréal, QC). The virus-containing supernatants were filtered through a 0.22 µm cellulose acetate syringe filter, ultracentrifugated and normalized for virion content using an in-house sensitive double-antibody sandwich enzyme-linked immunosorbent assay (ELISA) specific for the viral p24 protein [61].

### Cell culture

Purified human primary CD4^+^ T cells were isolated from peripheral blood mononuclear cells (PBMCs) using a negative selection kit according to the manufacturer's instructions (StemCell Technologies, Vancouver, BC). Cells were obtained from anonymous and paid, healthy volunteer donors that were specifically solicited for provision of these samples. Healthy subjects signed an informed consent approved by the Centre Hospitalier de l'Université Laval Institutional Review Board. These cells were either left untreated (to obtain quiescent cells) or activated with PHA-L (1 µg/ml) for 3 days prior their use (to obtain mitogen-stimulated cells) and maintained in complete RPMI-1640 culture medium supplemented with rhIL-2 (30 U/ml) at a density of 2×10^6^ cells/ml. Experiments were performed with cell preparations containing a minimal amount of contaminants as demonstrated previously (i.e. CD4^+^ T cells: purity >98%) [62].

### CD4^+^ T cells from HIV-1 patients

Patient samples were obtained from two aviremic HIV-1-infected patients that were undergoing antiretroviral therapy (kindly provided by Dr. Rafick-Pierre Sékaly, Université de Montréal, Montréal, QC) and also from three additional viremic and treatment-naive patients (kindly supplied by Dr. Jean-Pierre Routy at McGill University through the FRSQ - Réseau SIDA et Maladies Infectieuses) [63]. Purification of CD4^+^ T cells was achieved using magnetic beads as described above. The first aviremic donor had a CD4^+^ T cell count of 463/mm^3^ and was undergoing a combined antiretroviral therapy consisting of 3TC, EFV and abacavir. The second aviremic donor had a CD4^+^ T cell count of 499/mm^3^ and was treated with D4T and atazanavir. In both individuals, the viral load was undetectable (i.e. <50 copies/ml). The CD4^+^ T cell counts for viremic/untreated patients A, B and C were 290/mm^3^, 520/mm^3^ and 420/mm^3^ respectively, whereas their respective plasma viral loads were 224×10^3^, 172×10^3^ and 83×10^3^ HIV-1 RNA per ml.

### Ethics statement

Cells were obtained from anonymous and paid, healthy volunteer donors that were specifically solicited for provision of these samples. Healthy subjects signed an informed consent approved by the Centre Hospitalier de l'Université Laval Institutional Review Board. Patient samples were obtained from peripheral blood in accordance with the guidelines of the Institutional Bioethics Committee. All subjects signed an ethics board-approved informed consent form.

### HIV-1 infection of CD4^+^ T cells

In some experiments, purified CD4^+^ T cells (1×10^6^) were incubated for 2 h with NL4-3, Nef-deleted NL4-3, Vpr-deleted NL4-3, or NL4-3/Bal*env* (100 ng of p24). After three extensive washes with phosphate-buffered saline (PBS), the cells were cultured for 3 days in complete RPMI-1640 culture medium supplemented with rhIL-2 (30 U/ml), before staining and flow cytometry analysis. For other infection studies, CD4^+^ T cells were incubated for 2 or 3 days with NL4-3-IRES-HSA (100 ng p24/10^6^ cells), in the absence or presence of EFV (50 nM). Mock-infected cells were used as negative controls.

### Studies with cell-free supernatants

Purified CD4^+^ T cells (1×10^6^) were initially infected for 3 days with NL4-3. Next, supernatants from cells acutely infected with HIV-1 were filtrated and ultracentrifugated to eliminate cellular debris. Finally, cells were incubated with such cell-free supernatants and DCIR expression and apoptosis were monitored by flow cytometry. Controls consisted of cells incubated with cell-free supernatants from mock-infected cells.

### Flow cytometric analyses

Purified cells (1×10^6^) were incubated for 45 min at 4°C with a combination of antibodies made of either FITC-anti-DCIR (0.25 µg) and R-PE-anti-HSA (1 µg), R-PE-anti-DCIR (0.25 µg) and FITC-VAD-FMK (0.5 µg), or R-PE-anti-HSA (1 µg) and FITC-VAD-FMK (0.5 µg). Non-specific staining was assessed by using an isotype-matched irrelevant control Ab for DCIR (i.e. FITC- or R-PE-labeled IgG_1_) or mock-infected cells for HSA. Cells were then washed twice with PBS and 0.5% bovine serum albumin. Cells were fixed in 2% paraformaldehyde for 30 min at 4°C. Cell surface expression of DCIR and HSA was monitored using an Epics ELITE ESP apparatus (Coulter Electronics, Burlington, ON). Single stained cells were used as controls for compensation adjustments.

### Catalase assay

Purified CD4^+^ T cells (1×10^6^) were first pretreated with PEG-catalase (200 U/ml) (Sigma) for 10 min at 37°C. Next, cells were infected with NL4-3-IRES-HSA virions (100 ng of p24) and PEG-catalase was added to the culture medium every day. Flow cytometry analyses were performed to assess the percentage of cells positive for DCIR and FITC-VAD-FMK.

### Apoptosis studies

Apoptosis was induced by incubating resting or PHA-activated CD4^+^ T cells (1×10^6^ cells/ml) with different concentrations of H_2_O_2_ for increasing time lengths. Where indicated, apoptosis was induced by a treatment for 16 h at 37°C with the protein kinase C inhibitor staurosporine (1 µg/ml). The cell-permeable, FITC-conjugated, pan-caspase inhibitor FITC-VAD-FMK (R&D Systems) was used to detect activated caspases in CD4^+^ T cells by flow cytometry. Briefly, in a 24-well culture plate, cells (1×10^6^) in a final volume of 1 ml were stained directly with 10 µl of FITC-VAD-FMK and left at 37°C in the dark during the last 30 min of the apoptosis induction period. Cells were washed once in PBS to remove unbound reagent and fixed with paraformaldehyde or labeled with another Ab before flow cytometry analysis. Inhibition of apoptosis was achieved by pre-treating cells with Z-VAD-FMK (50 µM) (R&D Systems) for 1 h before H_2_O_2_ stimulation or HIV-1 pulsing. It is known that Z-VAD-FMK is an irreversible caspase inhibitor that binds to the active site of activated proteases and displays low cytotoxicity. Experiments aimed at studying the contribution of caspase-independent apoptotic pathway were performed using NAC (5 mM) from Sigma because this compound prevents nuclear translocation of AIF [64].

### HIV-1 binding/entry, infection and transfer experiments

Purified CD4^+^ T cells were treated with H_2_O_2_ (30 µM) during 16 h before performing the following experimental procedures. For the binding/entry assay, cells (1×10^6^) were incubated for 60 min at 37°C with NL4-3 (100 ng of p24). After three extensive washes with PBS to remove unabsorbed viruses, HIV-1 binding/entry was quantified by estimating the p24 content. For the infection assay, CD4^+^ T cells (1×10^6^) were incubated with NL4-3 (100 ng of p24) for 2 h. After three extensive washes with PBS, the cells were cultured in complete RPMI-1640 culture medium supplemented with rhIL-2 (30 U/ml). Virus production was estimated by assessing the p24 levels in cell-free culture supernatants. For the transfer study, CD4^+^ T cells (1×10^6^) were incubated with NL4-3 (100 ng of p24) for 2 h and after washes, autologous activated CD4^+^ T cells (1×10^6^) were added (ratio 1∶1) in complete RPMI-1640 culture medium supplemented with rhIL-2 (30 U/ml). Every two days, half of the medium was removed and kept frozen at −20°C and fresh medium was added to the culture. Virus production was estimated by measuring the p24 levels in cell-free culture supernatants. Virus transmission was also assessed using purified DCIR-negative and -positive cells. In brief, CD4^+^ T cells were first exposed to H_2_O_2_ to induce DCIR expression. Next, DCIR-negative and DCIR-positive cells were isolated and used separately in HIV-1 transfer experiments as described above. Cell isolation was achieved using the EasySep Biotin Selection kit according to the manufacturer's instructions with slight modifications (StemCell Technologies Inc., Vancouver, BC). The biotinylated anti-DCIR antibody (clone 216110 from R&D Systems) was used at a final concentration of 3 µg/ml. In some experiments, a dual staining technique was used to estimate the percentage of cells expressing surface DCIR and intracellular p24 by flow cytometry. Staining of the intracellular viral p24 core protein was achieved using the BD Cytofix/Cytoperm kit (BD Biosciences) and the monoclonal KC57 anti-p24 Ab (Beckman Coulter).

### Statistical analyses

Statistical analyses were carried out according to the methods outlined in Zar [65] and Sokal and Rohlf [66]. Means were compared using Student's t test. P values of less than 0.05 were deemed statistically significant. Calculations were performed with the GraphPad Prism software.
